# Dropsy Cured by Hydriodate of Potash

**Published:** 1834

**Authors:** 


9. *Dropsy cured by the Hydriodate of Potash.*—It appears from the London Medical and Surgical Journal, that Dr. Blake and

Mr. Hughes of England, have lately cured several inveterate cases of ascites and anasarca with this medicine. Some of them were apparently complicated with disease of the liver from intemperance. It operated as a diuretic, and is moreover thought by Mr. Hughes to have a specific and salutary action on the liver. In one case which is reported—

"The abdomen was èxceedingly large; the scrotum, legs, thighs, and arms, and integuments of the chest and back, were likewise very much swollen, so much so as to preclude the possibility of his bending the elbow to put his hand to his head. He then commenced taking the hydriodate of potass, in ten grain doses, three times a day, in a glass of water, gradually increasing the quantity to fifteen grains, which he continued to the 10th of March, with very marked benefit. The appetite and strength increased, and he was soon able to leave his bed and walk down stairs, owing to the decrease of the general swelling, which was accompanied by a proportionate increase in the quantity of urine. He omitted the hydriodate from the 10th to the 18th of March, in consequence of the intensely bitter taste he experienced, but the moment he did so the strength and appetite decreased, while the other symptoms became worse. He then recommenced this medicine with equal good effects, and continued it up to the 30th of April, when he again omitted it, thinking himself so nearly well, and on account of the disagreeable taste it left in the mouth. He, however, again found it necessary to resume its use on the 18th of May, and continued it to the 1st of June, with progressive improvement in every respect."

